# Recent advances in understanding intestinal stem cell regulation

**DOI:** 10.12688/f1000research.16793.1

**Published:** 2019-01-18

**Authors:** Deqing Hu, Han Yan, Xi C He, Linheng Li

**Affiliations:** 1 Department of Cell Biology, 2011 Collaborative Innovation Center of Tianjin for Medical Epigenetics; Tianjin Key Laboratory of Medical Epigenetics, Tianjin Medical University, Heping, Tianjin, China; 2 Key Laboratory of Breast Cancer Prevention and Therapy, Ministry of Education, Tianjin's Clinical Research Center for Cancer, Tianjin Medical University Cancer Institute and Hospital, National Clinical Research Center for Cancer, Heping, Tianjin, China; 3 Stowers Institute for Medical Research, Kansas City, USA; 4 Department of Pathology & Laboratory Medicine, University of Kansas Medical Center, Kansas City, China

**Keywords:** Intestinal stem cell, Homeostasis, Regeneration, Plasticity, Epigenetics, Nich

## Abstract

Intestinal homeostasis and regeneration are driven by intestinal stem cells (ISCs) lying in the crypt. In addition to the actively cycling ISCs that maintain daily homeostasis, accumulating evidence supports the existence of other pools of stem/progenitor cells with the capacity to repair damaged tissue and facilitate rapid restoration of intestinal integrity after injuries. Appropriate control of ISCs and other populations of intestinal epithelial cells with stem cell activity is essential for intestinal homeostasis and regeneration while their deregulation is implicated in colorectal tumorigenesis. In this review, we will summarize the recent findings about ISC identity and cellular plasticity in intestine, discuss regulatory mechanisms that control ISCs for intestinal homeostasis and regeneration, and put a particular emphasis on extrinsic niche-derived signaling and intrinsic epigenetic regulation. Moreover, we highlight several fundamental questions about the precise mechanisms conferring robust capacity for intestine to maintain physiological homeostasis and repair injuries.

## Introduction

**Figure 1.**
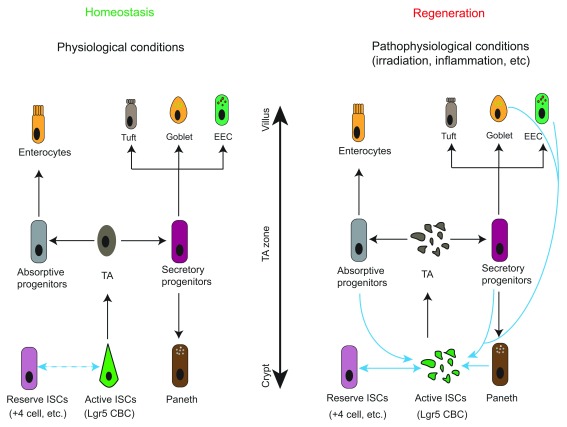
Hierarchy and plasticity of intestinal stem cells (ISCs). Under homeostatic conditions, active ISCs such as Lgr5 crypt base columnar cells (CBCs) migrate upwards to become transit-amplifying (TA) cells in the TA zone. TA cells divide rapidly and specify into either absorptive or secretory progenitors. Absorptive progenitors further differentiate into large quantities of enterocytes, while secretory progenitors commit to the Paneth, goblet, enteroendocrine (EEC), or goblet cells. Interconversion between reserve ISCs and active ISCs occurs occasionally in this setting (demonstrated by double-headed dash line). In response to radio- or chemo-therapies, the highly proliferative Lgr5 CBCs and TA cells are ablated. Reserve ISCs enter the cell cycle to replenish CBCs for subsequent regenerative process. Plasticity of differentiated progenies, including secretory and absorptive progenitors as well as terminally differentiated EEC and Paneth cells, has been observed when CBCs are damaged. These cells can revert to active ISCs and give rise to all intestinal cell types. However, whether they can bypass CBCs to transdifferentiate directly into other intestinal lineages and their functional importance to intestinal regeneration upon injuries remain to be determined.

Intestinal epithelium is one of the fastest renewing tissues in mammals. Owing to the constant exposure of its luminal surface to injurious factors, such as pathogens and toxins, the average turnover rate is every three to five days in mice to replenish damaged cells ^1^. These single-layered intestinal epithelial cells are organized into a crypt-villus structure and are predominantly composed of absorptive enterocytes and four secretory lineages known as enteroendocrine, Paneth, goblet, and tuft cells ( Figure 1). The thin intestinal epithelial sheet is responsible for nutrient absorption and stool compaction and also serves as a front-line barrier against microorganisms and infections ^2^. Maintenance and regeneration of this high-turnover tissue upon injury are fueled primarily by intestinal stem cells (ISCs) that reside at the bottom of the crypt, while differentiated absorptive and secretory cells are assembled as clusters or scattered along the crypt-villus axis ^3, 4^. More recently, considerable cellular plasticity was noted within intestine, and lineage-restricted progenitors or fully differentiated cells were able to replenish the tissue under certain conditions, such as DNA damage–induced injuries ^5– 11^.

ISCs possess both self-renewing capacity and multipotency to give rise to all types of intestinal epithelial cells. They divide and migrate upward to the middle region of the crypt to convert into transit-amplifying (TA) cells that divide rapidly for massive expansion before specializing into absorptive or secretory lineages ^4^. Owing to the tremendous regenerative capacity and the simple anatomic structure, intestine and particularly its ISCs have become an elegant model system for studying homeostasis, regeneration, and oncogenic transformation of mammalian adult tissues ^12, 13^. Self-renewal and multipotency of ISCs and plasticity of intestinal epithelial cells are largely controlled by external signals emanated from neighboring niche cells and intrinsic molecular processes, including epigenetic regulation ^14, 15^. In support of a central role of extracellular niche factors, ISCs embedded in the crypt bottom are sustained by signals emanated from both epithelial and mesenchymal niches. In the *in vitro* 3D culture system, ISCs are able to self-organize into crypt-villus–like structures referred to as “organoids” (or precisely enteroids or colonoids if derived from small intestine or colon, respectively) in the presence of a defined set of growth factors ^16^. These organoids comprise self-renewing ISCs intermingled with Paneth cells at the base of budding crypt and various differentiated lineages at blunt villus-like compartments and can be grown and maintained for many passages without losing normal karyotype over time ^17^.

In this review, we summarize the latest advances in our understanding of ISC identity, cellular plasticity, the basis for intestinal homeostasis and regeneration as well as how ISC self-renewal and multipotency are regulated, with a particular focus on extrinsic niche-derived signaling and intrinsically epigenetic regulation *.* Considering such progress in the mechanistic understanding of intestinal homeostasis and regeneration as well as the development of new models and techniques to faithfully mimic intestinal pathophysiology, we envision a variety of potent and effective therapeutic approaches for the treatment of intestinal diseases.

## Intestinal stem cells and cellular plasticity in intestine

For decades, crypts have been known as compartments comprising cellular sources for continuous intestinal homeostasis and robust post-injury regeneration ^18^. However, the cellular basis and nature of ISCs that fuel the rapid renewal of intestine have been among the mysteries in the field of adult stem cell biology. It has long been assumed that mammalian tissue-resident adult stem cells, including ISCs, predominantly reside out of the cell cycle in a relatively quiescent G _0_ state so that genomic integrity can be sustained in response to genotoxic insults ^2, 19^. However, this prevailing notion has been amended by the identification of long-lived yet rapidly dividing intestinal crypt base columnar cells (CBCs) with relatively specific expression of Lgr5 ^20^. They self-renew and are capable of differentiating into all types of intestinal epithelial cells in *in vivo*, as evidenced by lineage-tracing studies of *in vivo* and *in vitro* cultured organoids ^16, 20, 21^. Owing to their mitotically active feature, Lgr5 CBCs were termed active ISCs and thought to sustain physiological homeostasis of the rapid renewing intestine ^3^. Intriguingly, a subset of epithelial cells residing specifically at +4 position relative to the base of crypts was observed to share some properties of tissue-resident adult stem cells, such as the ability of long-term DNA label retention and a strong resistance to stress, including chemotherapy and irradiation ^19, 22, 23^, and thus had been postulated to represent ISCs long before Lgr5 CBCs were identified.

Lgr5 CBCs are mitotically active and can regenerate whole intestinal epithelium under homeostatic conditions ^20^. However, owing to their exquisite sensitivity to genotoxic stresses, Lgr5 CBCs are rapidly lost upon radio-/chemo-induced damage and thus could not account for the robust regenerative potential of post-injury intestine ^24^. Moreover, studies with genetic ablation of Lgr5 CBCs by diphtheria toxin (DT) treatment of mice harboring Lgr5-driven DT receptor (DTR) allele revealed that these cells are dispensable for normal intestinal homeostasis, implying the existence of other epithelial cells with both stem cell activity and DNA damage–resistant capacity to replace Lgr5 CBC loss for intestinal regeneration ^25^. Multiple populations of rare crypt cells marked by Bmi1 ^26^, Hopx ^26^, mTert ^27^, Krt19 ^28^, Lrig1 ^29^, Sox9 ^30^, Mex3a ^31^, or Prox1 ^6^ have been found to reside at approximately +4 position by short-term CreER-activated cell fate mapping assay. In sharp contrast to Lgr5 CBCs, most cells labeled by these reporter alleles are slowly cycling and injury-resistant and can give rise to clonal lineage-tracing events albeit at much lower frequency than Lgr5 CBCs ^5^. In light of the above features, these reporter-marked, predominantly +4 resident cells were defined as reserve ISCs in the literature ^3^.

In contrast to their unique spatial localization noted in genetic-marked reporter assays, transcriptomic analyses revealed that endogenous Bmi1, mTert, and Hopx are broadly expressed throughout crypt cells, even in the active Lgr5 CBCs, reflecting a certain inconsistency between reporter activity and actual mRNA expression of the endogenous alleles ^32– 34^. Multiple reasons could underlie this discrepancy, such as (1) difference in the 3′ untranslated region (UTR) sequence between CreER reporter and endogenous alleles. A direct comparison between the mRNA level of CreER reporter and endogenous alleles among distinct populations of crypt cells could determine whether CreER reporter can faithfully recapitulate expression of its endogenous counterpart at transcriptional and post-transcriptional levels. (2) As activation of genetic reporters in lineage-tracing studies requires reaching a certain threshold of CreER activity, cells marked by genetic reporters following short-term tamoxifen administration may point to a stronger CreER activity in these cells than in other populations of crypt cells, which could result from differential levels of CreER protein or tamoxifen permeability in distinct types of crypt cells. Ideally, development of immunohistological grade antibodies that can specially recognize the endogenous protein of reported markers for ISCs will resolve these discrepancies and help determine their actual distribution pattern throughout crypts.

As reserve ISCs marked by Bmi1 ^+^, Hopx ^+^, mTert, Lrig1, and so on can give rise to all types of epithelial lineages, including the active Lgr5 CBCs in lineage-tracing studies, they have initially been posited to sit at the apex of the cellular hierarchy in intestine ^25, 26^. However, single- or bulk-cell transcriptomic profiling analyses have invariably detected the expression of a few enteroendocrine markers within reserve populations of ISCs, indicating some common features between reserve ISCs and committed enteroendocrine cells or hinting at a potential developmental plasticity of lineage-restricted secretory progenitors in intestine ^35^. It should be mentioned that definition of reserve ISCs hinges solely on the functional criteria, including injury resistance, multipotency, cell cycle entry from G _0_ to G _1_ upon damage, and long-term maintenance, all of which can be illustrated by Cre recombinase–induced lineage-tracing assays ^3, 5^. Cells marked with Bmi1 ^+^, Hopx ^+^, mTert, and so on fulfill these functionally defining criteria and could act as bona fide reserve ISCs for tissue regeneration ^24, 26, 27, 36^. The fact that stem cells can express multiple-lineage genes in a fluctuating way, assumed as priming, has been reported in adult stem cells ^37^ as well as in pluripotent embryonic stem cells, which express a significant level of representative genes for primordial germ cells in an undifferentiated pluripotent state ^38– 40^. Therefore, caution should be exercised when defining ISC identity simply on the basis of transcriptomic analysis. Nevertheless, more intense studies to further investigate the heterogeneity of +4 epithelial cells with single-cell high-throughput strategies are required to deepen our understanding of the identity of reserve stem cells in intestine.

Cellular plasticity has been observed in several mammalian tissues and could act as an additional mechanism for tissue regeneration ^41^. Direct evidence showing developmental plasticity of enteroendocrine progenitors comes from lineage tracing of cells marked with Prox1, which is a transcription factor expressed predominantly in mature enteroendocrine cells and is essential for its commitment from ISCs ^6, 42– 45^. Prox1 enteroendocrine cells can function as reserve ISCs as they assume both homeostatic and injury-inducible stem activity in lineage-tracing reporter assays ^6^. Besides mature enteroendocrine cells, considerable plasticity and clonal lineage-tracing events have been noted in other progenies of Lgr5 CBCs, such as enteroendocrine progenitors marked by Bmi1 ^7^, Alpi ^+^ absorptive progenitors ^8^, secretory precursors expressing Atoh1 ^10^ or Dll1 ^11^, CD69 ^+^ CD274 ^+^ goblet precursor ^7^, fully differentiated Paneth cells ^46, 47^, and a small population of Dclk1 ^+^ tuft cells ^48^ in certain situations, such as inflammation, radio-/chemo-induced intestinal injuries, or upon loss of Lgr5 CBCs by DT administration ( Figure 1). However, the functional robustness and contribution of these lineage-committed cells to intestinal homeostasis and restoration upon tissue damage remain unclear and will be worth future investigation. Furthermore, whether these differentiated epithelia can bypass Lgr5 CBCs and transdifferentiate directly into other epithelial lineages is also instrumental for understanding the mechanisms of regenerative process and awaits clarification in forthcoming studies. Another concern to be considered is that haploinsufficiency in the knocked-in allele used in current genetic tracing assays was recently reported to result in a misleading phenotype as seen in pancreatic systems ^49^.

## Extrinsic niche regulation of intestinal stem cells

The activity of ISCs is stringently controlled to ensure proper proliferation and differentiation. Tight regulation of ISCs is achieved primarily through extrinsic signaling molecules emanated from their surrounding cells that altogether constitute a unique niche microenvironment ^15, 50^. Recent intensive studies have led to gradual identification of vital niche components that include both epithelial progenies of ISCs and mesenchymal cells, such as Paneth cells, fibroblasts, immune cells, enteric neurons, and endothelial cells ^51, 52^. When perturbed by injurious factors, these intestinal niche cells can be rewired for coordinated production of cytokines and growth factors to activate ISCs for rapid regeneration. To date, various niche factor–derived signaling pathways have been identified to be essential for ISC activity, intestinal homeostasis, and regeneration. In this section, we will briefly summarize the central roles of Wnt, bone morphogenetic protein (BMP), Notch, epidermal growth factor (EGF), and Hippo signaling in ISC regulation.

In the Wnt pathway, binding of Wnt ligands to their Frizzled receptor on targeted cells induces collapse of cytoplasmic APC destruction complex and subsequent nuclear translocation of β-catenin to activate Wnt target genes through association with T-cell factor (TCF) transcription factors ^53^. Wnt signaling is progressively reduced from crypt to villus axis and is indispensable for ISC maintenance and intestinal regeneration ^32, 54, 55^. Its abrogation via inactivating Tcf1/2 mutation ^56^, Tcf4 deletion ^57^, or exogenous expression of Wnt inhibitor Dkk1 ^58, 59^ invariably leads to Lgr5 CBC loss and decreased crypt cell proliferation. Augmentation of activity of this pathway by Apc inactivating mutation, constitutive activating mutation in β-catenin, or simultaneous deletion of two E3 ligases (Rnf43 and Znrf3) targeting Wnt ligand receptors for degradation unanimously result in crypt expansion and rapid appearance of intestinal adenomas in mice ^60– 64^. Although the functional importance of Wnt signaling and its contribution to intestinal regulation have been well appreciated ^55^, the cellular source for Wnt ligands remains incompletely understood. Various types of niche cells have been found to express a significant level of Wnt ligands, such as small intestinal Paneth cells ^51^, colonic Reg4 ^+^^65^ or cKit ^+^^66^ secretory cells, and numerous subsets of stromal cells ^67, 68^. Although epithelial Wnts can promote expansion of Lgr5 CBCs during *in vitro* organoid culture, their ablation does not demonstrate a notable impact on crypt cell proliferation and intestinal homeostasis *in vivo*, suggesting a significant redundancy of cellular origin of Wnt ligands in sustaining intestinal integrity ^67– 70^. In line with this assumption, recent studies found that Foxl1 ^+^^71, 72^, Pdgfrα ^+^^73^, Gli1 ^+^^55, 74^, and CD34 ^+^ Gp38 ^+^ αSMA ^–^^75^ mesenchymal cells serve as major sources of Wnt activity and play pivotal roles in sustaining intestinal homeostasis *in vivo*. Abolition of Wnt secretion in Foxl1 ^+^, Gli1 ^+^, or Pdgfrα ^+^ cells by genetic excision of porcupine ( *Porcn*) impairs proliferation of Lgr5 CBCs and leads to corruption of intestinal integrity ^71, 73, 74^. Whether these mesenchymal cells with distinct markers overlap with each other to some extent or even represent the same subsets of non-epithelial cells is currently unclear. Moreover, how Wnt activity in these mesenchymal cells is regulated in response to intestinal injuries, such as inflammation and chemo-/radio-toxicity, remains unanswered. Future investigations combining genetic approaches with single-cell RNA-sequencing technology will address these critical questions.

BMP signaling assumes an increasing gradient along the intestinal crypt-villus axis and serves as a critical inducer for ISC differentiation, thereby playing a vital role in balancing the effect of Wnt signaling on intestinal homeostasis ^76, 77^. BMPs belong to the transforming growth factor-beta superfamily of ligands and can induce phosphorylation of cytoplasmic R-Smads (Smad1/5/8) through binding to the membrane-embedded serine/threonine kinase type I and II receptors. Phosphorylated R-Smads form a complex with Co-Smad (Smad4) and subsequently enter the nucleus, where they regulate expression of targeted genes through association with a variety of co-factors ^78, 79^. The initial experimental evidence implicating an inhibitory role of BMP signaling in ISC self-renewal comes from conditional deletion of BMP receptor *Bmpr1a* and transgenic expression of BMP antagonist *Noggin* in mice, both of which unanimously lead to development of multiple polyposis in small intestine ^80– 82^. Similarly, aberrant expression of BMP antagonist Gremlin1 was noted in patients with hereditary mixed polyposis syndrome and its transgenic expression in mice leads to the appearance of ectopic crypts and subsequent oncogenic transformation ^83, 84^. Gene expression and *in situ* hybridization analyses demonstrated that BMP ligands and BMP antagonists are produced primarily by mesenchymes residing at different regions of the crypt-villus unit. BMP2 and BMP4 are secreted by intravillus and intercrypt mesenchymal cells, whereas Noggin, Gremlin1, Gremlin2, and chordin-like 1 antagonists are expressed by cryptal myofibroblasts and smooth muscle cells beneath the crypt bottom ^80, 85^. To date, little is known about the role and cellular source of other BMP ligands in intestine and whether ablation of these niche cells has an impact on intestinal integrity and tumorigenesis. Future studies are needed to address questions in this regard.

Notch receptors are single-pass type I transmembrane heterodimer proteins that comprise functional extracellular truncation, transmembrane, and intracellular (NICD) domain. Activation of Notch receptors begins with their binding to Notch ligands presented on an adjacent cell, which leads to cytoplasmic proteolytic cleavage of Notch receptors by γ-secretase complex and subsequent nuclear translocation of intracellular NICD to regulate transcription of target genes ^86^. Notch signaling has been shown to contribute to Lgr5 CBC maintenance and proliferation ^87– 89^ and its inhibition skews intestinal differentiation toward goblet lineage ^90– 92^. Paneth cells express and present Notch ligands Dll1 and Dll4 to adjacent Lgr5 CBCs to facilitate their self-renewal ^51^. Moreover, activation of Notch signaling in Paneth cells by forced expression of NICD can result in acquisition of a stem-like property in these cells ^46^. Notch signaling is also involved in controlling secretory and absorptive lineage determination when CBCs start to differentiate. Its activation stimulates Hes1 expression for transcriptional silencing of Atoh1, a master transcription factor for secretory lineage determination, thereby blocking secretory differentiation but promoting enterocyte specification of Notch receptor activated cells ^93^. Expression of Notch ligand Dll1 on secretory cells prevents a secretory fate of neighboring cells but drives these cells toward absorptive lineage through Notch signaling activation, which is a biological event referred to as lateral inhibition ^88^. Although Notch signaling contributes to CBC proliferation and intestinal homeostasis, the role of niche cells expressing Notch ligands remains unclear.

The Hippo pathway functions as a vital regulator of tissue homeostasis and organ size and its deregulation is implicated in the development of multiple types of cancer in humans. In the presence of extracellular stimuli, transcriptional activator YAP/TAZ complex is phosphorylated by upstream serine–threonine kinase MST1/MST2/Sav1-LATS1/2/MOB1A/B cascade, leading to either cytoplasmic sequestration or proteasomal degradation of YAP/TAZ. Without Hippo activation, non-phosphorylated YAP/TAZ enters the nucleus and acts as a co-activator for the TEAD transcription factor to regulate expression of genes associated with cell growth and proliferation ^94, 95^. The Hippo pathway is constitutively active in intestine under homeostatic conditions and its genetic inactivation via transgenic expression of YAP/TAZ or conditional deletion of Sav1, an upstream negative regulator of YAP, increases ISC proliferation and leads to crypt hyperplasia ^96, 97^. Hippo signaling is also indispensable for intestinal regeneration in mouse models of dextran sodium sulfate (DSS)-induced colitis and radiation-induced injury ^97, 98^. Transgenic expression of YAP induces an EGF signaling–dependent regenerative program to facilitate intestinal regeneration ^98^. Appropriate activation of Wnt signaling is essential for intestinal recovery following injury. However, its aberrant activation could lead to oncogenic transformation or reduce ISC survival through increasing radio-sensitivity of ISCs to DNA damage during intestinal regeneration ^99^. In addition to the transcriptional regulatory activity of nuclear YAP/TAP complex, unphosphorylated cytoplasmic complexes are integral components of β-catenin destruction complex and thus play a critical role in restricting Wnt signaling activity ^100^. Paradoxically, it has been noted that conditional deletion of YAP in intestinal epithelia augments Wnt activity and causes crypt hyperplasia and overgrowth throughout small intestine and colon after radiation-induced injury ^101^. These findings suggest that the Hippo pathway has dual roles in regulating both Wnt and Egf signaling and its impact on intestinal regeneration is context-dependent and could be determined by net activity of these two pathways. More work is needed to deepen our understanding of the molecular mechanisms that control the balanced activation of Wnt and Egf pathways by Hippo signaling during intestinal regeneration.

EGF communicates with target cells through the EGF receptor, which is a member of the ErbB family of tyrosine receptor kinases ^102^. Paneth cells express EGF and sustain proliferation of Lgr5 CBCs through its receptor ErbB ^103^. EGF supplementation of culture medium dramatically increases the efficiency of organoid formation *in vitro* ^17^. Lrig1 is a negative feedback regulator of ErbB receptor and its ablation causes crypt expansion in mice ^29, 104^. Although these findings support a crucial role of EGF signaling in CBC proliferation, the additional cellular source of EGF growth factor except for Paneth cells in intestine remains ambiguous.

## Intrinsically epigenetic regulation of intestinal stem cells

Intestinal homeostasis and regeneration are accompanied by drastic transcriptional alterations that are achieved through the cooperation between extrinsic niche signaling–controlled transcription factors/co-activators and intrinsic epigenetic regulators. Epigenetics refers to the inherited alteration in gene expression and phenotype that occur without changes in DNA sequence. It consists primarily of DNA methylation, histone modification, and chromatin remodeling ^105, 106^. In contrast to the extensively studied extrinsic niche factors, the role of intrinsically epigenetic mechanisms in intestinal homeostasis and regeneration is poorly understood. In this section, we will briefly discuss the currently limited knowledge of epigenetic mechanisms in intestinal regulation.

**Figure 2.**
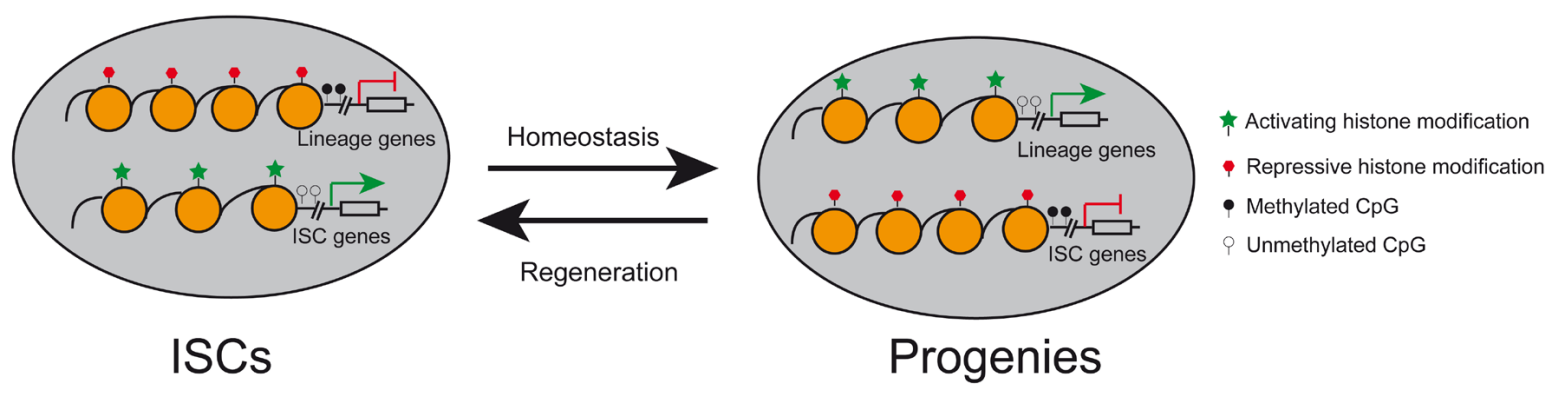
Epigenetic reprogramming for intestinal homeostasis and regeneration. During homeostasis, intestinal stem cells (ISCs) adopt a unique epigenetic signature and chromatin accessibility that collaboratively lead to expression of ISC-related genes and shutdown of lineage-specific factors. Lineage specification of ISCs is accompanied by epigenetic remodeling and chromatin accessibility changes that turn off ISCs genes while activating lineage-restricted genes. During the regenerative process, epigenetic landscape and chromatin accessibility are reconfigured to resemble ISCs during dedifferentiation of ISC progenies.

With the tremendous advancements in fluorescence-activated cell sorting, identification of lineage-specific markers, and next-generation sequencing technology, epigenetic characterization of distinct types of intestinal epithelial cells has recently become feasible. Initial studies with DNase I mapping for accessible chromatin regions and H3K4me2 and H3K27ac ChIP-seq (chromatin immunoprecipitation sequencing) for active enhancers showed that ISCs and enterocyte and secretory progenitors assume remarkable similarity in distribution pattern of open chromatin elements and active enhancers throughout the genome ^107^. However, these observations are not in line with the subsequent studies that employ assay for transposase-accessible chromatin using sequencing (ATAC-seq) to profile open chromatin regions among ISCs and enterocyte and secretory progenitors. The latter studies identified thousands of unique open chromatin regions among these populations and found that accessible chromatin states in secretory progenitors shift to resemble ISCs during dedifferentiation upon injuries ( Figure 2) ^7^. Although the discrepancy between the two studies can be explained by cellular heterogeneity and the different sensitivity of technologies employed, the functional importance of those unique open chromatin elements in secretory lineage specification, dedifferentiation, and regeneration has not been determined experimentally. In addition, comprehensive profiling of distinct histone modifications in purer populations of intestinal lineages will determine whether the epigenetic landscape varies during intestinal homeostasis and regeneration.

Initial bisulfite-sequencing of ISCs and their differentiated progenies in villus revealed minimal dynamics of DNA methylome during intestinal differentiation ^108^. This study used a cutoff of a minimal differential methylation of 40% to identify differentially methylated regions (DMRs) between ISCs and villous cells. As the distal regulatory regions are low-methylated at an overall methylation level of 30% in intestinal cells and display average change of about 15% in DNA methylation during intestinal differentiation, the cutoff criteria of analysis used in the study eliminates the identification of DMRs during differentiation of ISCs ^108, 109^. Subsequent bisulfite-sequencing studies with distinct analytic standards identified many DMRs at enhancers and disclosed a tight correlation between the DMRs and transcriptional alterations between two populations of cells, implying that the DNA methylation may have a vital role in intestinal renewal ^110^. This viewpoint has been proven by genetic ablation of *Dnmt1* and *Tet1* in intestine, which are maintenance methyltransferase and hydroxylase for DNA methylation, respectively ^106^. Dnmt1 is essential for intestinal development in newborn mice as inducible deletion of *Dnmt1* at perinatal stage causes genomic instability, premature differentiation, apoptosis, loss of villi, and decreased proliferation of crypt cells ^111, 112^. The essential role of DNA methylation for intestinal development is also reflected in Tet1-deleted mice. Tet1-null mice exhibit retarded growth, shorter intestines, weaker capability to form *in vitro* organoids, and reduced postnatal viability ^113^. Acute ablation of *Dnmt1* in adult intestinal epithelia causes a slight expansion of the proliferative crypt zone. However the crypt morphology recovers and DNA methylation restores to normal level several days after *Dnmt1* deficiency. A subsequent study revealed that *de novo* methyltransferase *Dnmt3b* can compensate for *Dnmt1* loss to maintain intestinal integrity as ablation of both enzymes leads to genome demethylation, genomic instability, increased apoptosis, and decreased survival ^114^. Taken together, these pieces of genetic evidence strongly support that dynamic regulation of DNA methylation acts as an essential epigenetic mechanism underlying intestinal development and homeostasis.

Post-translational modification of histones, such as methylation and ubiquitination, constitutes an additional component of epigenetic mechanism in transcriptional regulation, embryonic development, and adult tissue homeostasis ^115^. Monoubiquitination of lysine 119 on H2A (H2A119 mUb) and methylation of lysine 27 on H3 (H3K27me) correlate with transcriptional repression and are implemented by polycomb repressive complex 1 (PRC1) and polycomb repressive complex 2 (PRC2), respectively ^116^. The activity of PRC1 and PRC2 stems from Ring1a/1b E3 ligase and Ezh1/2 methyltransferases and has been shown to be essential for normal intestinal homeostasis or regeneration following injuries. Loss of function of the total PRC1 activity via *Ring1a/b* double deletion in intestinal epithelia compromises ISC self-renewal and intestinal integrity and results in morbidity through de-repression of a number of transcription factors that negatively regulate Wnt signaling ^117^.

Loss of PRC2 activity via Ah ^Cre^ or VillinCreER-induced epithelial deletion of the Eed, a scaffold protein of the complex, leads to a clear defect in cell proliferation in crypts, a marked increase in the number of goblet cells, mislocalized Paneth cells, and compromised regenerative capacity ^118– 120^. The homeostatic and regenerative defect of intestine in the absence of PRC2 results at least in part from aberrant upregulation of Cdkn2a and some master regulators for secretory lineages that are bivalently marked, normally targeted, and repressed by this complex ^118^. Conditional ablation of catalytic subunit of PRC2 complex Ezh2 does not show any abnormalities in the intestinal homeostasis, indicating that the other H3K27 methyltransferase Ezh1 could compensate for Ezh2 loss to maintain intestinal integrity ^120^. Further studies are needed to know the role and target genes of individual non-canonic PRC1 and PRC2 complexes in intestinal renewal and regeneration.

## Conclusions and future perspectives

Over the past decade, remarkable progress has been made in our understanding of intestinal biology. It has become evident that active Lgr5 CBCs and reserve ISCs work in a coordinated manner to maintain intestinal homeostasis and replenish the tissue upon injuries. In addition, differentiated epithelial cells possess considerable plasticity and can dedifferentiate to ISCs for intestinal regeneration in mouse models when active CBCs are damaged or artificially removed. Many niche-derived factors and a few epigenetic regulators have been identified and characterized for their roles in intestinal morphogenesis, homeostasis, and regeneration. Despite these considerable advances in recent years, several fundamental questions remain to be addressed about the precise molecular mechanisms controlling self-renewal, lineage commitment, and plasticity of ISCs. First, the cellular source of distinct environmental factors crucial for ISC function and the role of immune cells, peripheral nerve cells, and other ISC-proximal cells remain poorly understood; thus, extensive studies of these niche components will offer more insight into the extrinsic regulatory mechanisms for ISC function. Second, comprehensive characterization of the epigenetic landscape of various intestinal cells coupled with conditional ablation of key chromatin regulators in murine models will help determine the molecular basis underlying the remarkable plasticity of intestine in response to injuries. Third, the physiological function and contribution of differentiated crypt cells to radio-/chemo-induced intestinal regeneration remain to be experimentally evaluated. Fourth, the molecular mechanisms that reserve ISCs and differentiated crypt cells use to sense and replenish lost CBCs are largely unexplored; future investigation in this regard will help design effective therapeutic approaches to facilitate intestinal recovery after damage. Lastly, does aging-associated loss of regenerative potential of ISCs result from alterations in niche factors or epigenetic regulation or both ^121– 123^? The more we understand the above fundamental questions, the better we can employ ISCs for regenerative medicine.

## References

[1] Beumer J Clevers H: Regulation and plasticity of intestinal stem cells during homeostasis and regeneration. *Development.* 2016;143(20):3639–49. 10.1242/dev.133132 27802133

[2] Clevers H: The intestinal crypt, a prototype stem cell compartment. *Cell.* 2013;154(2):274–84. 10.1016/j.cell.2013.07.004 23870119

[3] Li L Clevers H: Coexistence of quiescent and active adult stem cells in mammals. *Science.* 2010;327(5965):542–5. 10.1126/science.1180794 20110496 PMC4105182

[4] Barker N van de Wetering M Clevers H: The intestinal stem cell. *Genes Dev.* 2008;22(14):1856–64. 10.1101/gad.1674008 18628392 PMC2735277

[5] Yousefi M Li L Lengner CJ: Hierarchy and Plasticity in the Intestinal Stem Cell Compartment. *Trends Cell Biol.* 2017;27(10):753–64. 10.1016/j.tcb.2017.06.006 28732600 PMC5612891

[6] Yan KS Gevaert O Zheng GXY: Intestinal Enteroendocrine Lineage Cells Possess Homeostatic and Injury-Inducible Stem Cell Activity. *Cell Stem Cell.* 2017;21(1):78–90.e6. 10.1016/j.stem.2017.06.014 28686870 PMC5642297

[7] Jadhav U Saxena M O'Neill NK: Dynamic Reorganization of Chromatin Accessibility Signatures during Dedifferentiation of Secretory Precursors into Lgr5+ Intestinal Stem Cells. *Cell Stem Cell.* 2017;21(1):65–77.e5. 10.1016/j.stem.2017.05.001 28648363 PMC5505276

[8] Tetteh PW Basak O Farin HF: Replacement of Lost *Lgr5*-Positive Stem Cells through Plasticity of Their Enterocyte-Lineage Daughters. *Cell Stem Cell.* 2016;18(2):203–13. 10.1016/j.stem.2016.01.001 26831517

[9] Ishibashi F Shimizu H Nakata T: Contribution of ATOH1 ^+^ Cells to the Homeostasis, Repair, and Tumorigenesis of the Colonic Epithelium. *Stem Cell Reports.* 2018;10(1):27–42. 10.1016/j.stemcr.2017.11.006 29233556 PMC5768891

[10] Tomic G Morrissey E Kozar S: Phospho-regulation of ATOH1 Is Required for Plasticity of Secretory Progenitors and Tissue Regeneration. *Cell Stem Cell.* 2018;23(3):436–443.e7. 10.1016/j.stem.2018.07.002 30100168 PMC6138952

[11] van Es JH Sato T van de Wetering M: Dll1 ^+^ secretory progenitor cells revert to stem cells upon crypt damage. *Nat Cell Biol.* 2012;14(10):1099–104. 10.1038/ncb2581 23000963 PMC3789123

[12] Potten CS Booth C Hargreaves D: The small intestine as a model for evaluating adult tissue stem cell drug targets. *Cell Prolif.* 2003;36(3):115–29. 10.1046/j.1365-2184.2003.00264.x 12814429 PMC6496932

[13] Wang F Scoville D He XC: Isolation and characterization of intestinal stem cells based on surface marker combinations and colony-formation assay. *Gastroenterology.* 2013;145(2):383–395.e21. 10.1053/j.gastro.2013.04.050 23644405 PMC3781924

[14] Roostaee A Benoit YD Boudjadi S: Epigenetics in Intestinal Epithelial Cell Renewal. *J Cell Physiol.* 2016;231(11):2361–7. 10.1002/jcp.25401 27061836 PMC5074234

[15] Sailaja BS He XC Li L: The regulatory niche of intestinal stem cells. *J Physiol.* 2016;594(17):4827–36. 10.1113/JP271931 27060879 PMC5009778

[16] Sato T Vries RG Snippert HJ: Single Lgr5 stem cells build crypt-villus structures *in vitro* without a mesenchymal niche. *Nature.* 2009;459(7244):262–5. 10.1038/nature07935 19329995

[17] Sato T Clevers H: Growing self-organizing mini-guts from a single intestinal stem cell: mechanism and applications. *Science.* 2013;340(6137):1190–4. 10.1126/science.1234852 23744940

[18] Bjerknes M Cheng H: Intestinal epithelial stem cells and progenitors. *Methods Enzymol.* 2006;419:337–383. 10.1016/S0076-6879(06)19014-X 17141062

[19] Potten CS Kovacs L Hamilton E: Continuous labelling studies on mouse skin and intestine. *Cell Prolif.* 1974;7(3):271–83. 10.1111/j.1365-2184.1974.tb00907.x 4837676

[20] Barker N van Es JH Kuipers J: Identification of stem cells in small intestine and colon by marker gene *Lgr5*. *Nature.* 2007;449(7165):1003–7. 10.1038/nature06196 17934449

[21] Barker N van Es JH Jaks V: Very long-term self-renewal of small intestine, colon, and hair follicles from cycling *Lgr5* ^+ve^ stem cells. *Cold Spring Harb Symp Quant Biol.* 2008;73:351–6. 10.1101/sqb.2008.72.003 19478326

[22] Potten CS Owen G Booth D: Intestinal stem cells protect their genome by selective segregation of template DNA strands. *J Cell Sci.* 2002;115(Pt 11):2381–8. 12006622 10.1242/jcs.115.11.2381

[23] Potten CS: Extreme sensitivity of some intestinal crypt cells to X and gamma irradiation. *Nature.* 1977;269(5628):518–21. 10.1038/269518a0 909602

[24] Yan KS Chia LA Li X: The intestinal stem cell markers Bmi1 and Lgr5 identify two functionally distinct populations. *Proc Natl Acad Sci U S A.* 2012;109(2):466–71. 10.1073/pnas.1118857109 22190486 PMC3258636

[25] Tian H Biehs B Warming S: A reserve stem cell population in small intestine renders *Lgr5*-positive cells dispensable. *Nature.* 2011;478(7368):255–9. 10.1038/nature10408 21927002 PMC4251967

[26] Takeda N Jain R LeBoeuf MR: Interconversion between intestinal stem cell populations in distinct niches. *Science.* 2011;334(6061):1420–4. 10.1126/science.1213214 22075725 PMC3705713

[27] Montgomery RK Carlone DL Richmond CA: Mouse telomerase reverse transcriptase (mTert) expression marks slowly cycling intestinal stem cells. *Proc Natl Acad Sci U S A.* 2011;108(1):179–84. 10.1073/pnas.1013004108 21173232 PMC3017192

[28] Asfaha S Hayakawa Y Muley A: *Krt19*+/ *Lgr5*− Cells Are Radioresistant Cancer-Initiating Stem Cells in the Colon and Intestine. *Cell Stem Cell.* 2015;16(6):627–38. 10.1016/j.stem.2015.04.013 26046762 PMC4457942

[29] Powell AE Wang Y Li Y: The pan-ErbB negative regulator Lrig1 is an intestinal stem cell marker that functions as a tumor suppressor. *Cell.* 2012;149(1):146–58. 10.1016/j.cell.2012.02.042 22464327 PMC3563328

[30] Roche KC Gracz AD Liu XF: SOX9 maintains reserve stem cells and preserves radioresistance in mouse small intestine. *Gastroenterology.* 2015;149(6):1553–1563.e10. 10.1053/j.gastro.2015.07.004 26170137 PMC4709179

[31] Barriga FM Montagni E Mana M: Mex3a Marks a Slowly Dividing Subpopulation of Lgr5+ Intestinal Stem Cells. *Cell Stem Cell.* 2017;20(6):801–816.e7. 10.1016/j.stem.2017.02.007 28285904 PMC5774992

[32] Muñoz J Stange DE Schepers AG: The Lgr5 intestinal stem cell signature: robust expression of proposed quiescent '+4' cell markers. *EMBO J.* 2012;31(14):3079–91. 10.1038/emboj.2012.166 22692129 PMC3400017

[33] Itzkovitz S Lyubimova A Blat IC: Single-molecule transcript counting of stem-cell markers in the mouse intestine. *Nat Cell Biol.* 2011;14(1):106–14. 10.1038/ncb2384 22119784 PMC3292866

[34] Li N Yousefi M Nakauka-Ddamba A: Single-cell analysis of proxy reporter allele-marked epithelial cells establishes intestinal stem cell hierarchy. *Stem Cell Reports.* 2014;3(5):876–91. 10.1016/j.stemcr.2014.09.011 25418730 PMC4235148

[35] Buczacki SJ Zecchini HI Nicholson AM: Intestinal label-retaining cells are secretory precursors expressing Lgr5. *Nature.* 2013;495(7439):65–9. 10.1038/nature11965 23446353

[36] Sangiorgi E Capecchi MR: *Bmi1* is expressed *in vivo* in intestinal stem cells. *Nat Genet.* 2008;40(7):915–20. 10.1038/ng.165 18536716 PMC2906135

[37] Akashi K He X Chen J: Transcriptional accessibility for genes of multiple tissues and hematopoietic lineages is hierarchically controlled during early hematopoiesis. *Blood.* 2003;101(2):383–9. 10.1182/blood-2002-06-1780 12393558

[38] Yamaji M Ueda J Hayashi K: PRDM14 ensures naive pluripotency through dual regulation of signaling and epigenetic pathways in mouse embryonic stem cells. *Cell Stem Cell.* 2013;12(3):368–82. 10.1016/j.stem.2012.12.012 23333148

[39] Bao S Leitch HG Gillich A: The germ cell determinant Blimp1 is not required for derivation of pluripotent stem cells. *Cell Stem Cell.* 2012;11(1):110–7. 10.1016/j.stem.2012.02.023 22770244 PMC3391686

[40] Chu LF Surani MA Jaenisch R: *Blimp1* expression predicts embryonic stem cell development *in vitro*. *Curr Biol.* 2011;21(20):1759–65. 10.1016/j.cub.2011.09.010 22000107 PMC3203992

[41] Tetteh PW Farin HF Clevers H: Plasticity within stem cell hierarchies in mammalian epithelia. *Trends Cell Biol.* 2015;25(2):100–8. 10.1016/j.tcb.2014.09.003 25308311

[42] Petrova TV Nykänen A Norrmén C: Transcription factor PROX1 induces colon cancer progression by promoting the transition from benign to highly dysplastic phenotype. *Cancer Cell.* 2008;13(5):407–19. 10.1016/j.ccr.2008.02.020 18455124

[43] Egerod KL Engelstoft MS Grunddal KV: A major lineage of enteroendocrine cells coexpress CCK, secretin, GIP, GLP-1, PYY, and neurotensin but not somatostatin. *Endocrinology.* 2012;153(12):5782–95. 10.1210/en.2012-1595 23064014 PMC7958714

[44] Engelstoft MS Egerod KL Lund ML: Enteroendocrine cell types revisited. *Curr Opin Pharmacol.* 2013;13(6):912–21. 10.1016/j.coph.2013.09.018 24140256

[45] Habib AM Richards P Cairns LS: Overlap of endocrine hormone expression in the mouse intestine revealed by transcriptional profiling and flow cytometry. *Endocrinology.* 2012;153(7):3054–65. 10.1210/en.2011-2170 22685263 PMC3440453

[46] Yu S Tong K Zhao Y: Paneth Cell Multipotency Induced by Notch Activation following Injury. *Cell Stem Cell.* 2018;23(1):46–59.e5. 10.1016/j.stem.2018.05.002 29887318 PMC6035085

[47] Schmitt M Schewe M Sacchetti A: Paneth Cells Respond to Inflammation and Contribute to Tissue Regeneration by Acquiring Stem-like Features through SCF/c-Kit Signaling. *Cell Rep.* 2018;24(9):2312–2328.e7. 10.1016/j.celrep.2018.07.085 30157426

[48] Westphalen CB Asfaha S Hayakawa Y: Long-lived intestinal tuft cells serve as colon cancer-initiating cells. *J Clin Invest.* 2014;124(3):1283–95. 10.1172/JCI73434 24487592 PMC3934168

[49] Shen J Ha DP Zhu G: GRP78 haploinsufficiency suppresses acinar-to-ductal metaplasia, signaling, and mutant *Kras* -driven pancreatic tumorigenesis in mice. *Proc Natl Acad Sci.* 2017;114:E4020–E4029. 10.1073/pnas.1616060114 28461470 PMC5441757

[50] Chacón-Martínez CA Koester J Wickström SA: Signaling in the stem cell niche: regulating cell fate, function and plasticity. *Development.* 2018;145(15): pii: dev165399. 10.1242/dev.165399 30068689

[51] Sato T van Es JH Snippert HJ: Paneth cells constitute the niche for Lgr5 stem cells in intestinal crypts. *Nature.* 2011;469(7330):415–8. 10.1038/nature09637 21113151 PMC3547360

[52] Roulis M Flavell RA: Fibroblasts and myofibroblasts of the intestinal lamina propria in physiology and disease. *Differentiation.* 2016;92(3):116–31. 10.1016/j.diff.2016.05.002 27165847

[53] Clevers H Nusse R: Wnt/β-catenin signaling and disease. *Cell.* 2012;149(6):1192–205. 10.1016/j.cell.2012.05.012 22682243

[54] Batlle E Henderson JT Beghtel H: Beta-catenin and TCF mediate cell positioning in the intestinal epithelium by controlling the expression of EphB/ephrinB. *Cell.* 2002;111(2):251–63. 10.1016/S0092-8674%2802%2901015-2 12408869

[55] Valenta T Degirmenci B Moor AE: Wnt Ligands Secreted by Subepithelial Mesenchymal Cells Are Essential for the Survival of Intestinal Stem Cells and Gut Homeostasis. *Cell Rep.* 2016;15(5):911–8. 10.1016/j.celrep.2016.03.088 27117411

[56] Korinek V Barker N Willert K: Two members of the Tcf family implicated in Wnt/beta-catenin signaling during embryogenesis in the mouse. *Mol Cell Biol.* 1998;18(3):1248–56. 10.1128/MCB.18.3.1248 9488439 PMC108837

[57] van Es JH Haegebarth A Kujala P: A critical role for the Wnt effector Tcf4 in adult intestinal homeostatic self-renewal. *Mol Cell Biol.* 2012;32(10):1918–27. 10.1128/MCB.06288-11 22393260 PMC3347420

[58] Kuhnert F Davis CR Wang HT: Essential requirement for Wnt signaling in proliferation of adult small intestine and colon revealed by adenoviral expression of Dickkopf-1. *Proc Natl Acad Sci U S A.* 2004;101(1):266–71. 10.1073/pnas.2536800100 14695885 PMC314174

[59] Pinto D Gregorieff A Begthel H: Canonical Wnt signals are essential for homeostasis of the intestinal epithelium. *Genes Dev.* 2003;17(14):1709–13. 10.1101/gad.267103 12865297 PMC196179

[60] Koo BK Spit M Jordens I: Tumour suppressor RNF43 is a stem-cell E3 ligase that induces endocytosis of Wnt receptors. *Nature.* 2012;488(7413):665–9. 10.1038/nature11308 22895187

[61] Hao HX Xie Y Zhang Y: ZNRF3 promotes Wnt receptor turnover in an R-spondin-sensitive manner. *Nature.* 2012;485(7397):195–200. 10.1038/nature11019 22575959

[62] Jiang X Charlat O Zamponi R: Dishevelled promotes Wnt receptor degradation through recruitment of ZNRF3/RNF43 E3 ubiquitin ligases. *Mol Cell.* 2015;58(3):522–33. 10.1016/j.molcel.2015.03.015 25891077

[63] Korinek V Barker N Morin PJ: Constitutive transcriptional activation by a beta-catenin-Tcf complex in APC ^-/-^ colon carcinoma. *Science.* 1997;275(5307):1784–7. 10.1126/science.275.5307.1784 9065401

[64] Morin PJ Sparks AB Korinek V: Activation of beta-catenin-Tcf signaling in colon cancer by mutations in beta-catenin or APC. *Science.* 1997;275(5307):1787–90. 10.1126/science.275.5307.1787 9065402

[65] Sasaki N Sachs N Wiebrands K: Reg4 ^+^ deep crypt secretory cells function as epithelial niche for Lgr5 ^+^ stem cells in colon. *Proc Natl Acad Sci U S A.* 2016;113(37):E5399–407. 10.1073/pnas.1607327113 27573849 PMC5027439

[66] Rothenberg ME Nusse Y Kalisky T: Identification of a cKit ^+^ colonic crypt base secretory cell that supports Lgr5 ^+^ stem cells in mice. *Gastroenterology.* 2012;142(5):1195–1205.e6. 10.1053/j.gastro.2012.02.006 22333952 PMC3911891

[67] Farin HF van Es JH Clevers H: Redundant sources of Wnt regulate intestinal stem cells and promote formation of Paneth cells. *Gastroenterology.* 2012;143(6):1518–1529.e7. 10.1053/j.gastro.2012.08.031 22922422

[68] Kabiri Z Greicius G Madan B: Stroma provides an intestinal stem cell niche in the absence of epithelial Wnts. *Development.* 2014;141(11):2206–15. 10.1242/dev.104976 24821987

[69] Durand A Donahue B Peignon G: Functional intestinal stem cells after Paneth cell ablation induced by the loss of transcription factor Math1 (Atoh1). *Proc Natl Acad Sci U S A.* 2012;109(23):8965–70. 10.1073/pnas.1201652109 22586121 PMC3384132

[70] San Roman AK Jayewickreme CD Murtaugh LC: Wnt secretion from epithelial cells and subepithelial myofibroblasts is not required in the mouse intestinal stem cell niche *in vivo*. *Stem Cell Reports.* 2014;2(2):127–34. 10.1016/j.stemcr.2013.12.012 24527386 PMC3923227

[71] Shoshkes-Carmel M Wang YJ Wangensteen KJ: Subepithelial telocytes are an important source of Wnts that supports intestinal crypts. *Nature.* 2018;557(7704):242–6. 10.1038/s41586-018-0084-4 29720649 PMC5966331

[72] Aoki R Shoshkes-Carmel M Gao N: Foxl1-expressing mesenchymal cells constitute the intestinal stem cell niche. *Cell Mol Gastroenterol Hepatol.* 2016;2(2):175–88. 10.1016/j.jcmgh.2015.12.004 26949732 PMC4772878

[73] Greicius G Kabiri Z Sigmundsson K: *PDGFRα* ^+^ pericryptal stromal cells are the critical source of Wnts and RSPO3 for murine intestinal stem cells *in vivo*. *Proc Natl Acad Sci U S A.* 2018;115(14):E3173–E3181. 10.1073/pnas.1713510115 29559533 PMC5889626

[74] Degirmenci B Valenta T Dimitrieva S: GLI1-expressing mesenchymal cells form the essential Wnt-secreting niche for colon stem cells. *Nature.* 2018;558(7710):449–53. 10.1038/s41586-018-0190-3 29875413

[75] Stzepourginski I Nigro G Jacob JM: CD34+ mesenchymal cells are a major component of the intestinal stem cells niche at homeostasis and after injury. *Proc Natl Acad Sci U S A.* 2017;114(4):E506–E513. 10.1073/pnas.1620059114 28074039 PMC5278455

[76] Hardwick JCH van den Brink GR Bleuming SA: Bone morphogenetic protein 2 is expressed by, and acts upon, mature epithelial cells in the colon. *Gastroenterology.* 2004;126(1):111–21. 10.1053/j.gastro.2003.10.067 14699493

[77] Qi Z Li Y Zhao B: BMP restricts stemness of intestinal Lgr5 ^+^ stem cells by directly suppressing their signature genes. *Nat Commun.* 2017;8:13824. 10.1038/ncomms13824 28059064 PMC5227110

[78] David CJ Massagué J: Contextual determinants of TGFβ action in development, immunity and cancer. *Nat Rev Mol Cell Biol.* 2018;19(7):419–35. 10.1038/s41580-018-0007-0 29643418 PMC7457231

[79] Feng XH Derynck R: Specificity and versatility in tgf-beta signaling through Smads. *Annu Rev Cell Dev Biol.* 2005;21:659–93. 10.1146/annurev.cellbio.21.022404.142018 16212511

[80] He XC Zhang J Tong WG: BMP signaling inhibits intestinal stem cell self-renewal through suppression of Wnt-beta-catenin signaling. *Nat Genet.* 2004;36(10):1117–21. 10.1038/ng1430 15378062

[81] Haramis AP Begthel H van den Born M: *De novo* crypt formation and juvenile polyposis on BMP inhibition in mouse intestine. *Science.* 2004;303(5664):1684–6. 10.1126/science.1093587 15017003

[82] Batts LE Polk DB Dubois RN: Bmp signaling is required for intestinal growth and morphogenesis. *Dev Dyn.* 2006;235(6):1563–70. 10.1002/dvdy.20741 16538672

[83] Davis H Irshad S Bansal M: Aberrant epithelial *GREM1* expression initiates colonic tumorigenesis from cells outside the stem cell niche. *Nat Med.* 2015;21(1):62–70. 10.1038/nm.3750 25419707 PMC4594755

[84] Jaeger E Leedham S Lewis A: Hereditary mixed polyposis syndrome is caused by a 40-kb upstream duplication that leads to increased and ectopic expression of the BMP antagonist *GREM1*. *Nat Genet.* 2012;44(6):699–703. 10.1038/ng.2263 22561515 PMC4594751

[85] Kosinski C Li VS Chan AS: Gene expression patterns of human colon tops and basal crypts and BMP antagonists as intestinal stem cell niche factors. *Proc Natl Acad Sci U S A.* 2007;104(39):15418–23. 10.1073/pnas.0707210104 17881565 PMC2000506

[86] Kopan R Ilagan MX: The canonical Notch signaling pathway: unfolding the activation mechanism. *Cell.* 2009;137(2):216–33. 10.1016/j.cell.2009.03.045 19379690 PMC2827930

[87] Pellegrinet L Rodilla V Liu Z: Dll1- and dll4-mediated notch signaling are required for homeostasis of intestinal stem cells. *Gastroenterology.* 2011;140(4):1230–1240.e1-7. 10.1053/j.gastro.2011.01.005 21238454 PMC3066401

[88] VanDussen KL Carulli AJ Keeley TM: Notch signaling modulates proliferation and differentiation of intestinal crypt base columnar stem cells. *Development.* 2012;139(3):488–97. 10.1242/dev.070763 22190634 PMC3252352

[89] Tian H Biehs B Chiu C: Opposing activities of Notch and Wnt signaling regulate intestinal stem cells and gut homeostasis. *Cell Rep.* 2015;11(1):33–42. 10.1016/j.celrep.2015.03.007 25818302 PMC4394041

[90] Milano J McKay J Dagenais C: Modulation of notch processing by gamma-secretase inhibitors causes intestinal goblet cell metaplasia and induction of genes known to specify gut secretory lineage differentiation. *Toxicol Sci.* 2004;82(1):341–58. 10.1093/toxsci/kfh254 15319485

[91] van Es JH van Gijn ME Riccio O: Notch/gamma-secretase inhibition turns proliferative cells in intestinal crypts and adenomas into goblet cells. *Nature.* 2005;435(7044):959–63. 10.1038/nature03659 15959515

[92] van Es JH de Geest N van de Born M: Intestinal stem cells lacking the Math1 tumour suppressor are refractory to Notch inhibitors. *Nat Commun.* 2010;1(1):18. 10.1038/ncomms1017 20975679 PMC2895507

[93] Ueo T Imayoshi I Kobayashi T: The role of Hes genes in intestinal development, homeostasis and tumor formation. *Development.* 2012;139(6):1071–82. 10.1242/dev.069070 22318232

[94] Fu V Plouffe SW Guan KL: The Hippo pathway in organ development, homeostasis, and regeneration. *Curr Opin Cell Biol.* 2017;49:99–107. 10.1016/j.ceb.2017.12.012 29316535 PMC6348871

[95] Pan D: The hippo signaling pathway in development and cancer. *Dev Cell.* 2010;19(4):491–505. 10.1016/j.devcel.2010.09.011 20951342 PMC3124840

[96] Imajo M Ebisuya M Nishida E: Dual role of YAP and TAZ in renewal of the intestinal epithelium. *Nat Cell Biol.* 2015;17(1):7–19. 10.1038/ncb3084 25531778

[97] Cai J Zhang N Zheng Y: The Hippo signaling pathway restricts the oncogenic potential of an intestinal regeneration program. *Genes Dev.* 2010;24(21):2383–8. 10.1101/gad.1978810 21041407 PMC2964748

[98] Gregorieff A Liu Y Inanlou MR: Yap-dependent reprogramming of Lgr5 ^+^ stem cells drives intestinal regeneration and cancer. *Nature.* 2015;526(7575):715–8. 10.1038/nature15382 26503053

[99] Tao S Tang D Morita Y: Wnt activity and basal niche position sensitize intestinal stem and progenitor cells to DNA damage. *EMBO J.* 2015;34(5):624–40. 10.15252/embj.201490700 25609789 PMC4365032

[100] Azzolin L Panciera T Soligo S: YAP/TAZ incorporation in the β-catenin destruction complex orchestrates the Wnt response. *Cell.* 2014;158(1):157–70. 10.1016/j.cell.2014.06.013 24976009

[101] Barry ER Morikawa T Butler BL: Restriction of intestinal stem cell expansion and the regenerative response by YAP. *Nature.* 2013;493(7430):106–10. 10.1038/nature11693 23178811 PMC3536889

[102] Roskoski R Jr: The ErbB/HER family of protein-tyrosine kinases and cancer. *Pharmacol Res.* 2014;79:34–74. 10.1016/j.phrs.2013.11.002 24269963

[103] Schepers A Clevers H: Wnt signaling, stem cells, and cancer of the gastrointestinal tract. *Cold Spring Harb Perspect Biol.* 2012;4(4):a007989. 10.1101/cshperspect.a007989 22474007 PMC3312683

[104] Wong VW Stange DE Page ME: Lrig1 controls intestinal stem-cell homeostasis by negative regulation of ErbB signalling. *Nat Cell Biol.* 2012;14(4):401–8. 10.1038/ncb2464 22388892 PMC3378643

[105] Allis CD Jenuwein T: The molecular hallmarks of epigenetic control. *Nat Rev Genet.* 2016;17(8):487–500. 10.1038/nrg.2016.59 27346641

[106] Hu D Shilatifard A: Epigenetics of hematopoiesis and hematological malignancies. *Genes Dev.* 2016;30(18):2021–41. 10.1101/gad.284109.116 27798847 PMC5066610

[107] Kim TH Li F Ferreiro-Neira I: Broadly permissive intestinal chromatin underlies lateral inhibition and cell plasticity. *Nature.* 2014;506(7489):511–5. 10.1038/nature12903 24413398 PMC4151315

[108] Kaaij LT van de Wetering M Fang F: DNA methylation dynamics during intestinal stem cell differentiation reveals enhancers driving gene expression in the villus. *Genome Biol.* 2013;14(5):R50. 10.1186/gb-2013-14-5-r50 23714178 PMC4053812

[109] Stadler MB Murr R Burger L: DNA-binding factors shape the mouse methylome at distal regulatory regions. *Nature.* 2011;480(7378):490–5. 10.1038/nature10716 22170606

[110] Sheaffer KL Kim R Aoki R: DNA methylation is required for the control of stem cell differentiation in the small intestine. *Genes Dev.* 2014;28(6):652–64. 10.1101/gad.230318.113 24637118 PMC3967052

[111] Elliott EN Sheaffer KL Schug J: Dnmt1 is essential to maintain progenitors in the perinatal intestinal epithelium. *Development.* 2015;142(12):2163–72. 10.1242/dev.117341 26023099 PMC4483766

[112] Yu DH Gadkari M Zhou Q: Postnatal epigenetic regulation of intestinal stem cells requires DNA methylation and is guided by the microbiome. *Genome Biol.* 2015;16:211. 10.1186/s13059-015-0763-5 26420038 PMC4589031

[113] Kim R Sheaffer KL Choi I: Epigenetic regulation of intestinal stem cells by Tet1-mediated DNA hydroxymethylation. *Genes Dev.* 2016;30(21):2433–42. 10.1101/gad.288035.116 27856615 PMC5131782

[114] Elliott EN Sheaffer KL Kaestner KH: The ' *de novo*' DNA methyltransferase Dnmt3b compensates the Dnmt1-deficient intestinal epithelium. *eLife.* 2016;5: pii: e12975. 10.7554/eLife.12975 26808831 PMC4786433

[115] Shilatifard A: The COMPASS family of histone H3K4 methylases: mechanisms of regulation in development and disease pathogenesis. *Annu Rev Biochem.* 2012;81:65–95. 10.1146/annurev-biochem-051710-134100 22663077 PMC4010150

[116] Margueron R Reinberg D: The Polycomb complex PRC2 and its mark in life. *Nature.* 2011;469(7330):343–9. 10.1038/nature09784 21248841 PMC3760771

[117] Chiacchiera F Rossi A Jammula S: Polycomb Complex PRC1 Preserves Intestinal Stem Cell Identity by Sustaining Wnt/β-Catenin Transcriptional Activity. *Cell Stem Cell.* 2016;18(1):91–103. 10.1016/j.stem.2015.09.019 26526724

[118] Chiacchiera F Rossi A Jammula S: PRC2 preserves intestinal progenitors and restricts secretory lineage commitment. *EMBO J.* 2016;35(21):2301–14. 10.15252/embj.201694550 27585866 PMC5090213

[119] Jadhav U Nalapareddy K Saxena M: Acquired Tissue-Specific Promoter Bivalency Is a Basis for PRC2 Necessity in Adult Cells. *Cell.* 2016;165(6):1389–400. 10.1016/j.cell.2016.04.031 27212235 PMC4893000

[120] Koppens MA Bounova G Gargiulo G: Deletion of Polycomb Repressive Complex 2 From Mouse Intestine Causes Loss of Stem Cells. *Gastroenterology.* 2016;151(4):684–697.e12. 10.1053/j.gastro.2016.06.020 27342214

[121] Mihaylova MM Cheng CW Cao AQ: Fasting Activates Fatty Acid Oxidation to Enhance Intestinal Stem Cell Function during Homeostasis and Aging. *Cell Stem Cell.* 2018;22(5):769–778.e4. 10.1016/j.stem.2018.04.001 29727683 PMC5940005

[122] Potten CS Martin K Kirkwood TB: Ageing of murine small intestinal stem cells. *Novartis Found Symp.* 2001;235:66–79; discussion 79–84, 101–4. 10.1002/0470868694.ch7 11280034

[123] Nalapareddy K Nattamai KJ Kumar RS: Canonical Wnt Signaling Ameliorates Aging of Intestinal Stem Cells. *Cell Rep.* 2017;18(11):2608–21. 10.1016/j.celrep.2017.02.056 28297666 PMC5987258

